# Controlling heterologous protein synthesis through a plant RNA ThermoSwitch

**DOI:** 10.1186/s13007-026-01517-6

**Published:** 2026-03-30

**Authors:** Filip Lastovka, Hadrien Peyret, George P. Lomonossoff, Betty Y.-W. Chung

**Affiliations:** 1 Department of Pathology, University of Cambridge, Tennis Court Road, Cambridge, CB2 1QP UK; 2 Department of Biochemistry and Metabolism, John Innes Centre, Norwich Research Park, Norwich, NR4 7UH UK; 3 Division of Plant and Crop Sciences, Sutton Bonnington Campus, University of Nottingham, Sutton Bonington, Loughborough, LE12 5RD UK

## Abstract

**Background:**

Plants are far from passive in the face of changing temperatures and have evolved transcriptional, post-transcriptional, and post-translational strategies to stay one step ahead of the environment. Among the most exciting recent discoveries are RNA ThermoSwitches, embedded within 5′ untranslated regions (UTR) of several mRNAs. These *cis-*acting RNA elements sense temperature shifts and instantly tune translational output, acting as molecular thermostats inside the cell. First uncovered in *Arabidopsis thaliana*, ThermoSwitches are now emerging as a powerful new frontier for biotechnology, offering a programmable way to modulate protein production through temperature adjustments.

**Results:**

Using a dual fluorescence reporter construct introduced by *Agrobacterium*-mediated transient expression, we have demonstrated that the native ThermoSwitch within the 5′ UTR of the *Arabidopsis* phytochrome-interacting factor 7 (*PIF7*) mRNA functions as a potent temperature-responsive module in *Nicotiana benthamiana* leaves. A shift from 17 to 27 °C triggered a pronounced increase in reporter translation, delivering ~ 70% higher output within one day. This magnitude of enhancement mirrors the rise in endogenous *PIF7* translation observed in *Arabidopsis* following the same temperature shift. The response remained even when the native upstream 5′ UTR context was replaced with an unrelated sequence, demonstrating that the ThermoSwitch operates autonomously. Crucially, locking the hairpin into a strengthened, rigid conformation abolished the temperature response entirely.

**Conclusions:**

This work provides the first in vivo evidence that a native plant RNA ThermoSwitch functions effectively in an *Agrobacterium*-mediated transient expression system, establishing a homogeneous, temperature-responsive gene regulation system, free from chemical inducers or repressors. Demonstrating that the *Arabidopsis* PIF7 ThermoSwitch operates autonomously in *Nicotiana benthamiana* highlights its value as a versatile plug-and-play module that can be readily deployed across plant systems for broad biotechnological applications.

**Supplementary Information:**

The online version contains supplementary material available at 10.1186/s13007-026-01517-6.

## Background

Plants have evolved mechanisms to adapt to fluctuations in surrounding temperatures, to which they are constantly exposed. Many such mechanisms operating at transcriptional, post-transcriptional, and post-translational levels have been extensively investigated [1, 2]. Recent studies have revealed the existence of a direct translational control mechanism that operates *in cis* in several plant mRNAs. Eukaryotic RNA ThermoSwitches, first discovered in *Arabidopsis thaliana*, act as temperature sensors to directly regulate protein synthesis from the associated mRNAs [3–5].

**Fig. 1:**
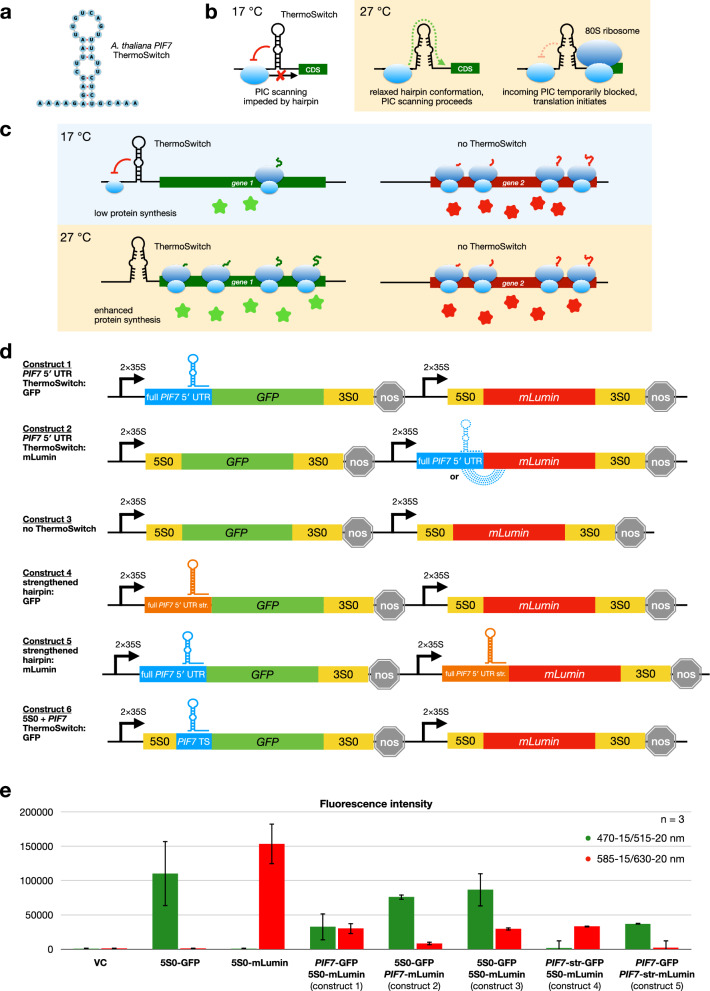
**a** The *Arabidopsis thaliana PIF7* ThermoSwitch hairpin visualized using *forna* [18]. **b** and **c** Regulation of protein synthesis by the ThermoSwitch before and after a temperature shift. At 17 °C, the stable ThermoSwitch hairpin likely inhibits translation by impeding the scanning pre-initiation complex. At 27 °C, the ThermoSwitch adopts a relaxed conformation, which allows easier unwinding and progression of the pre-initiation complex to the translation initiation site, resulting in more protein synthesized. The relaxed hairpin may contribute to efficient initiation by temporarily blocking the incoming pre-initiation complex. Unstructured 5′ UTR does not impede translation initiation at low temperatures. Translation initiation factors have been omitted from the illustration for simplicity. **d** Illustration of the plasmid-borne reporter constructs used in this study. The backbones of the constructs, which are excluded from the illustration, are identical and based on pHRE [10]. The reporter genes along with a *Tomato zonate spot virus* post-transcriptional silencing suppressor gene and a kanamycin resistance gene are located in the T-DNA region of the plasmid. 2 × 35S = double *Cauliflower mosaic virus* 35S promoter. *nos* = nopaline synthase gene 3′ regulatory region (‘terminator’). 5S0 and 3S0 = synthetic 5′ and 3′ UTRs of pHRE [10]. *PIF7* 5′ UTR str. = strengthened hairpin with all non-GC base pairing changed to GC base pairing and closed mid-stem bulge through GC base pairing. Alternative base pairing of the ThermoSwitch sequence with the *mLumin* CDS in construct 2 (see Figure S1c) is shown in dotted blue lines. **e** Fluorometry measurement of the clarified soluble fraction produced from three discs extracted from each area of *N. benthamiana* leaves five days post infiltration and growth in a glasshouse (without temperature shift). Red (585-15/630-20 nm) and green (470-15/515-20 nm) fluorescence signal is coloured red and green, respectively.

One of the best characterized plant RNA ThermoSwitches is an mRNA hairpin within the 5′ UTR of the transcription factor gene *PIF7* (phytochrome-interacting factor 7; Fig. 1a). The *PIF7* RNA ThermoSwitch is located 25 nt upstream of the translation start site and directly modulates synthesis of the PIF7 protein in response to temperature. Experiments in vitro have demonstrated that below 22 °C, the RNA ThermoSwitch is in a stable hairpin conformation that likely impedes the scanning pre-initiation complex (PIC) from accessing the translation initiation site (Fig. 1b). When the temperature rises to 27 °C (and up to 32 °C), the hairpin relaxes, allowing the PIC to continue scanning to the initiation site for protein synthesis [3, 4]. Concurrently, the refolded ThermoSwitch may transiently obstruct the incoming PIC, thereby facilitating initiation by the preceding complex. The PIF7 protein subsequently drives the transcription of growth-related genes, including the auxin biosynthesis gene cluster, thereby mediating daytime rhythmic growth and development of the plant in response to the environmental temperature [3, 4]. In part because of the inherently different translational initiation processes of prokaryotes and eukaryotes, eukaryotic RNA ThermoSwitches are distinct from bacterial RNA thermometers, which gradually melt at higher temperatures or switch to an alternative conformation, revealing or obscuring the prokaryotic ribosome-binding site [4–6].

Precision tuning of protein production in plants through a controlled shift in growth temperature is potentially a powerful and versatile tool for biotechnology, especially in circumstances where constitutive overproduction is detrimental. Here, we provide the first in vivo demonstration that a plant RNA ThermoSwitch can deliver such temperature-sensitive control of gene expression when deployed via *Agrobacterium*-mediated transient expression (Fig. 1c).

## Results

The *PIF7* 5′ UTR ThermoSwitch was assessed in vivo in a transient expression context by incorporating it into fluorescence reporter constructs introduced into *Nicotiana benthamiana* via *Agrobacterium tumefaciens*. Reporter output was compared following a temperature shift from 17 °C to 27 °C. Although both circular dichroism and fluorescence resonance energy transfer confirmed that the ThermoSwitch undergoes conformation changes in vitro between 22 °C and 27 °C [3], 17 °C was chosen as the lower temperature to ensure stable hairpin structure and to minimize the interference of potential temperature fluctuations during the experiment.

The investigation faced two complicating factors. Firstly, as with most biochemical reactions, translation rate increases with temperature, which requires a correction for the overall rise in protein synthesis after the temperature shift [7, 8]. Secondly, metabolic activity varies among leaves and even among different regions on the same leaf, with the sampled area being the greatest source of variation in transient expression, followed by the position of the leaf on the plant [9]. To address these issues, a dual reporter system was implemented in the pHRE expression vector [10]. This design allows ThermoSwitch-dependent expression to be normalized against a control reporter gene placed downstream of an artificially engineered linear 5′ UTR (5S0). The transcription of both reporter genes, *GFP* and *mLumin*, was driven by identical promoters, and they were positioned on the same T-DNA (Fig. 1d), ensuring that equal number of copies of each reporter gene were delivered to the same area of an infiltrated leaf. As a result, both signals reflected the metabolic activity of the same cells and were equally affected by the general increase in expression at elevated temperature.

Several additional features of the system were evaluated. Firstly, the ability to generate distinct, non-overlapping fluorescence signals measurable above background was confirmed in *Agrobacterium*-infiltrated plants grown in a glasshouse (Figs. 1e, S1a and b). As expected, the ThermoSwitch-containing reporter produced a weaker signal (GFP signal reduced to 43% and mLumin signal reduced to 27%) than the 5S0-containing reporter , which was optimized for high expression levels [10]. When mLumin was compared across constructs, the same gene driven by the same promoter produced a weaker signal when placed downstream of *GFP* in the dual-reporter configuration than in a single-reporter construct, consistently with positional effects previously reported in *Agrobacterium*-mediated expression systems [11, 12]. Importantly, *mLumin* expression with the 5S0 5′ UTR was not significantly influenced by different levels of *GFP* expression in constructs carrying alternative 5′ UTRs and neither were *GFP* expression levels affected by the different *mLumin* 5′ UTRs. Furthermore, potential base pairing between the ThermoSwitch and the *mLumin* coding sequence led to the selection of *GFP* as the reporter to assess ThermoSwitch-controlled expression (figure S1c).

**Fig. 2:**
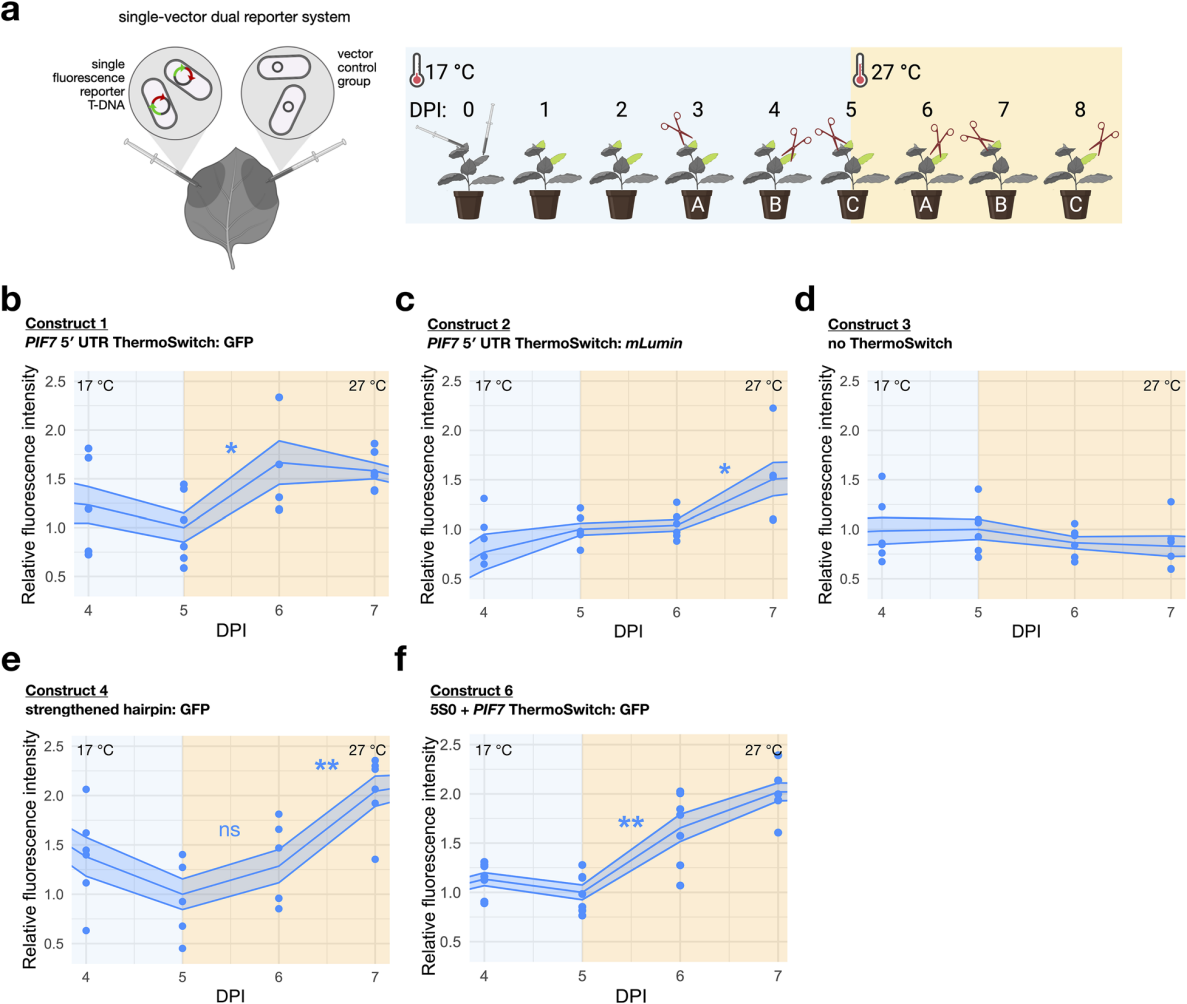
**a** Infiltration of *Agrobacterium* suspensions into *N. benthamiana* leaves and the time course experiment. The dual reporter construct and the empty vector (pHRE) were introduced into separate areas on the leaf. The infiltrated leaves are highlighted in green. The temperature was shifted from 17 °C to 27 °C following the harvest on day five post infiltration. Scissors indicate leaf harvest, which rotated among three groups (A, B, C) of plants. The subfigure was created in BioRender. Lastovka, F. (2026) https://BioRender.com/d1fkxq3. **b** Green fluorescence intensity (*PIF7* full ThermoSwitch-containing UTR) normalized to the red fluorescence intensity (5S0, without any ThermoSwitch) and represented relatively to the mean value at 5 DPI to allow easier comparison of expression shifts among experiments. The asterisk represents a significant difference following the temperature shift: *P* = 0.035, *n* = 6, Welch’s *t* test. The orange area represents a temperature of 27 °C. The ribbon shows the mean and the SEM. Differences between adjacent time points without an asterisk were not statistically significant. **c** Relative red fluorescence intensity—PIF7 full, but alternative secondary structure—normalized to the green fluorescence intensity—5S0. No significant difference following the temperature shift between time points 5 and 6 DPI (*P* = 0.64), but the increase in relative fluorescence intensity on day 7 was significant: *P* = 0.037, *n* = 6, Welch’s *t* test. **d** Relative green fluorescence intensity—5S0—normalized to the red fluorescence intensity—an identical 5S0. No significant difference between adjacent time points. *n* = 6. **e** Relative green fluorescence intensity—PIF7 with strengthened hairpin—normalized to the red fluorescence intensity—5S0. No significant difference following the temperature shift between time points 5 and 6 DPI (*P* = 0.21), but the difference between time points 6 and 7 DPI was significant: *P* = 0.0076. *n* = 6. **f** Relative green fluorescence intensity—5S0 + ThermoSwitch—normalized to the red fluorescence intensity—5S0. The difference following the temperature shift was statistically significant: *P* = 0.0023, *n* = 7, Welch’s *t* test.

The dual reporter construct 1 comprising a *PIF7*-5′-UTR-fused *GFP* (5′ reporter) and a 5S0-fused *mLumin* (3′ reporter) was introduced into *N. benthamiana* leaves via *Agrobacterium* infiltration. To monitor the background fluorescence of infiltrated tissue, the empty vector, pHRE, was introduced into a separate area on the leaf. The plants were maintained in a growth cabinet at 17 °C for five days prior to shift to 27 °C, after which tissue was harvested daily from 3 to 8 days post infiltration for fluorescence quantification (Fig. 2a). A significant increase in the ThermoSwitch-regulated GFP signal was apparent one day after the temperature shift (Figs. 2b and S2a). Normalization to the mLumin signal, which controlled for tissue metabolic activity, revealed a 1.7-fold increase in GFP fluorescence following the transfer to 27 °C, similar to the translational induction of *PIF7* in *Arabidopsis* in response to the same shift [3].

To ensure that GFP induction was specifically due to the ThermoSwitch rather than reporter context, four additional constructs were generated. In construct 2, the ThermoSwitch was placed upstream of the downstream reporter *mLumin* to test whether the effect depended on reporter position; in this configuration, however, the temperature response was abolished, likely due to the potential base pairing between the ThermoSwitch sequence and the *mLumin* coding sequence. This highlights the importance of assessing potential RNA base-pairing interactions during reporter design (Figs. 2c and S2b). Construct 3, in which both reporters carried identical 5S0 5′ UTRs, tested whether differences in the coding sequence could explain relative fluorescence changes, for example due to different transcript or protein turnover. As expected, no temperature-shift-dependent effect was observed (Figs. 2d and S2c). Construct 4 was designed to test whether enhanced structural stability of the ThermoSwitch would suppress temperature sensitivity. The hairpin was strengthened by mutating all non-GC base pairing to GC base pairing and closing of the mid-stem bulge, also through GC base pairing. The temperature-sensitive control of expression was abolished in this construct, in line with the results from previous ThermoSwitch experiments in vitro (Figs. 2e and S2d) [3]. A direct comparison of the functional ThermoSwitch and the strengthened hairpin in construct 5 was not feasible, because stabilizing the hairpin suppressed *mLumin* translation to levels indistinguishable from the vector control (Figure S2e).

Finally, when the 5′ UTR sequence upstream of the *PIF7* ThermoSwitch was replaced with the 5S0 sequence (in construct 6), the normalized fluorescence increased to 1.7 times the pre-shift level (Figs. 2f and S2f), a level similar to that found with construct 1, confirming that the ThermoSwitch hairpin itself is the key autonomous thermoregulator.

## Discussion

This work demonstrates that the *PIF7* ThermoSwitch enables temperature-sensitive regulation of gene expression in an *Agrobacterium*-mediated transient transformation system *in planta*. While this system is already established for high-yield protein production, it also offers a versatile platform for implementing dynamic regulation, such as temperature-dependence, much faster with significantly less complexity than stable transformation [13, 14]. Building on previous observations of ThermoSwitch activity in chromosomal genes of *Arabidopsis* [3], this work establishes the ThermoSwitch as an RNA-based module for plant synthetic biology. Its ability to impose precise and externally controlled regulation highlights its potential for applications ranging from plant-made pharmaceuticals to programmable metabolic engineering. A key advantage of thermosensitive regulation is its homogeneity and independence from chemical inducers or suppressors.

Using fluorescence reporters in *N. benthamiana*, we showed that the *PIF7* 5′ UTR ThermoSwitch exerted temperature-dependent control of expression following a shift from 17 to 27 °C. Fluorometric quantification normalized against a ThermoSwitch-independent reporter revealed an additional 70% increase in *GFP* expression above the general rise observed at higher temperature. Although the dynamic range is relatively narrow, it may be sufficient to mitigate the negative effects of expressing toxic or metabolically demanding genes. Moreover, GFP was produced in considerable amounts before the temperature shift, showing that the natural *PIF7* ThermoSwitch constricts and enhances expression rather than shutting it off and turning it on, which is also the case in its native context [3]. This proof-of-concept study paves the way to engineering ThermoSwitches with a more precise on/off control. Further refinement of ThermoSwitch sequences will also expand their dynamic range to tune their switching temperature to values better suited to plant molecular farming or other biotechnological applications.

Importantly, the ThermoSwitch hairpin remained effective when placed in a heterologous 5′ UTR, demonstrating its versatility and compatibility with existing expression systems. However, the three characterized *Arabidopsis* RNA ThermoSwitches (in *PIF7*, *HSFA2*, and *WRKY22*) naturally reside 17–30 nt upstream of their respective coding sequence [3]. This conserved spacing suggests that the ThermoSwitch function may depend on occupying a specific positional window within the 5′ UTR, where its structural dynamics can most effectively modulate translation initiation. Our results have emphasized the need to take into account the sequence context of ThermoSwitches in artificial systems, because potential base pairing of the ThermoSwitch sequence with flanking regions, including the gene of interest, can disrupt the secondary structure and interfere with the temperature-sensing function. This is an important consideration during the design of expression systems that utilize functional RNA domains in general [5, 15, 16]. When more ThermoSwitches are developed or trialled for use in transient expression systems, using an alternative ThermoSwitch will be an option to avoid misfolding without the necessity to modify the coding sequence. The compatibility of a particular sequence context with a specific ThermoSwitch can be predicted bioinformatically but will need to be verified experimentally.

Building on this work, we will investigate sequence variants of *PIF7* and additional ThermoSwitches, including the *Arabidopsis thaliana HSFA2* element, to map the design rules that govern temperature-dependent control [3]. In parallel, we will identify native *N. benthamiana* ThermoSwitches and test their behaviour across different temperature regimes, establishing a broader toolkit for thermally regulated expression in diverse plant systems.

## Conclusions

This study provides the first evidence that an RNA ThermoSwitch can function in an *Agrobacterium*-mediated transient expression system. Demonstrating temperature-dependent translational control in this widely used platform shows that a native plant ThermoSwitch can operate autonomously outside its endogenous context and remain effective in a heterologous host. This establishes a foundation for developing temperature-responsive expression systems that may support applications in molecular farming or in engineered crops where controlled, temperature-linked modulation of gene expression is advantageous [4].

## Methods

### Design of the reporter system

The *Agrobacterium*-mediated transient expression plasmid pHRE [10] was used as a backbone for the reporter constructs. The *GFP* reporter gene originated from pHRE-GFP [10]. The red fluorescence gene used for the reporter system was *mLumin* [17] inserted into pHRE at the *Bsa*I restriction sites. *PIF7*-derived 5′ UTRs were inserted into the constructs at the *Bsm*BI restriction sites, replacing the original 5S0 5′ UTR, but retaining the Kozak sequence. To generate the dual reporter construct, the *GFP* reporter gene—which consists of the double 35S promoter (*Cauliflower mosaic virus*), the 5′ UTR, the CDS, the 3S0 3′ UTR and the *nos* (nopaline synthase) 3′ regulatory region (‘terminator’)—was amplified with primers that produced *Sac*I restriction sites at each terminus (supplementary table). This gene was then inserted into the pHRE-derived *mLumin* reporter plasmid at the *Sac*I restriction site in the same orientation as *mLumin*. The two reporter genes in the resulting construct possessed identical promoters and 3′ UTRs (Fig. 1d).

A control *Agrobacterium* suspension was included in the experiments, which carried the empty pHRE vector and transferred a silencing suppressor and kanamycin resistance genes, but no fluorescence genes. This enabled confirmation that any autofluorescence from the tissue or the defence response to infiltration was not interpreted as the reporter signal.

### Plant growth and infiltration

*Agrobacterium tumefaciens* strain LBA4404 carrying a reporter plasmid or the empty pHRE vector was cultured overnight from single colonies in 20–30 mL of LB supplemented with 50 μg mL^−1^ of kanamycin and 50 μg mL^−1^ of rifampicin in a 250 mL conical flask with a spring coil at 28 °C and 220 rpm. The overnight culture was pelleted by centrifugation in 50 mL tubes at a speed of 3.6 krcf, and the supernatant was discarded. The pellet was resuspended and diluted in infiltration buffer to OD_600_ 0.4. The infiltration buffer consisted of 10 mM 2-(*N*-morpholino)ethanesulfonic acid (MES) pH 5.6, 10 mM MgCl_2_, and 100 μM acetosyringone. The suspensions were incubated at room temperature for more than 1 h before infiltration.

The *Agrobacterium* suspension was infiltrated using a needleless syringe into leaves of potted five-week-old pre-flowering *N. benthamiana* cultivar LAB. The outlines of the infiltrated areas were marked with a marker. Each leaf was infiltrated with the reporter suspension as well as the empty vector suspension in separate non-overlapping areas (Fig. 2a, left). The plants were grown in a growth cabinet (Sanyo, MLR-352H-PE) set to a constant temperature of 17 °C and a light cycle with an 8-h-long darkness and a 16-h-long maximal light intensity. On day five post infiltration (5 DPI) after the leaf harvest, the temperature was increased to 27 °C (Fig. 2a, right).

### Harvest and lysis of leaves

The plants were assigned into three groups, with individuals of each group evenly distributed in the growth cabinet. Starting three days post infiltration (DPI), a single leaf of all plants in a single group was harvested by severing it at the petiole. At each time point, equal number of younger and older leaves of the two infiltrated leaves in each plant were harvested. The harvest rotated to another group the following day (Fig. 2a, right).

From each infiltrated area, three discs were extracted using a cork borer with a 12 mm diameter. The discs were placed into a shatterproof 2 mL centrifuge tube, snap-frozen in liquid nitrogen, and stored at −80 °C. The samples were further processed after the end of the time course. A ceramic bead and 270 μL of phosphate-buffered saline (PBS) with cOmplete EDTA-free protease inhibitor (Roche) were added to each tube. The discs were lysed using an Omni Bead Ruptor 24 homogenizer (settings: 30 s, single cycle, and maximal speed of 4 m s^−1^). To obtain the soluble fraction, the lysate was clarified twice by centrifugation at 16 krcf in the cold room for 10 min.

### Fluorometry

Each clarified lysate was transferred in triplicate to wells of a 96-well opaque F-bottom black microplate, with 60 μL in each well. The fluorescence was measured on a CLARIOstar Plus microplate reader (BMG LABTECH) using the top optic and 50 flashes per well. The excitation/emission wavelengths used were 470-15/515-20 nm to detect GFP and 585-15/630-20 nm to detect mLumin. To enable cross-plate comparisons, the gain was adjusted, separately for each experiment, at approximately 90% capacity using the most fluorescent well with lysate from 8 DPI (last day of the time course) and used for fluorescence measurement in all plates.

To calculate the normalized fluorescence intensity values, fluorescence intensity (mean of the fluorometry measurements in three wells corrected by the blank signal from a PBS-containing well) was adjusted by subtracting the mean fluorescence intensity of the empty vector lysates at the matching time point. The adjusted value of the examined reporter was divided by the adjusted fluorescence intensity of the reference reporter separately for each sample. The statistical significance of change in relative fluorescence intensity in the following time point was determined using Welch’s two-sample two-sided *t* test. The normal distribution of the data was verified with the Shapiro–Wilk test.

## Supplementary Information

Supplementary Material 1: Supplementary Figure 1** a** and **b**, Leaves of *N. benthamiana* five days post infiltration and growth in a glasshouse (without temperature shift) photographed under UV light from the abaxial side and with white light from the adaxial side. VC = vector control group (pHRE). *PIF7*-str = *PIF7* 5′ UTR with a strengthened hairpin. Fluorescence measurement shown in Fig. 1e. **c**, Secondary structure of 60 nt of the 5′ UTR and 60 nt of the reporter CDS predicted by *ViennaRNA* *RNAfold *[19] at 17 °C and visualized using *forna* [18]. The ThermoSwitch hairpin sequence is coloured blue and the *GFP* or *mLumin* CDS is coloured green or red, respectively. Supplementary Figure 2. **a–d**, Blank-corrected fluorescence values of the leaf homogenate soluble fractions from the temperature shift experiments in *Nicotiana benthamiana*. Green bars show the 470-15/515-20 nm signal and red bars show the 585-15/630-20 nm signal. Black bars indicate the fluorescence of the area on the same leaf to which the empty vector was introduced. These values gave rise to the normalized fluorescence values in subfigures 2b–e. Mean of three technical replicates for each sample is shown. Blue and orange areas indicate growth at 17 °C or 27 °C, respectively, before the harvest. The temperature was shifted after the harvest on the fifth day. The fluorometry gain was adjusted individually for each experiment. **f**, Blank-corrected red (585-15/630-20 nm) fluorescence values of the leaf homogenate soluble fractions. Comparison of the vector control group (black) with the construct bearing strengthened hairpin upstream of *mLumin* (red). The ribbon shows the mean and the SEM. **g**, Blank-corrected fluorescence values of the leaf homogenate soluble fractions from the experiment using a combined 5S0 + ThermoSwitch 5′ UTR upstream of *GFP*. Black bars indicate the fluorescence of the area on the same leaf to which the empty vector was introduced. These values gave rise to the normalized fluorescence values in subfigure 2f. Mean of three technical replicates for each sample is shown.

Supplementary Material 2: Supplementary Table: 5′ UTR and primer sequences.

## Data Availability

All data supporting the findings of this study are available within the paper and its Supplementary Information.

## Abbreviations

DPI: Days post infiltration
GFP: Green fluorescent protein
MES: 2-(*N*-Morpholino)ethanesulfonic acid
PBS: Phosphate-buffered saline
PIC: Pre-initiation complex
PIF7: Phytochrome-interacting factor 7
UTR: Untranslated region
