# Supplementary figures and images for: Controlling heterologous protein synthesis through a plant RNA ThermoSwitch
Source: Plant Methods. 2026 Mar 30;22:61. doi: 10.1186/s13007-026-01517-6 (PMC13353011; doi:10.1186/s13007-026-01517-6)

a

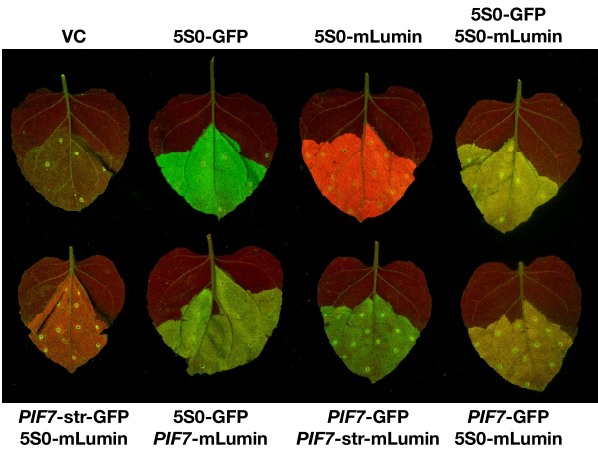

b

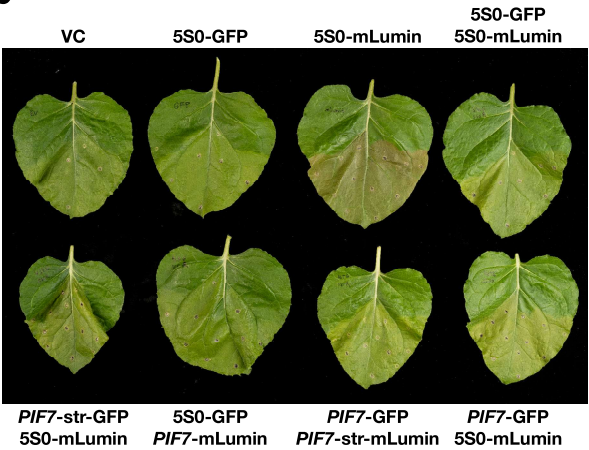

c

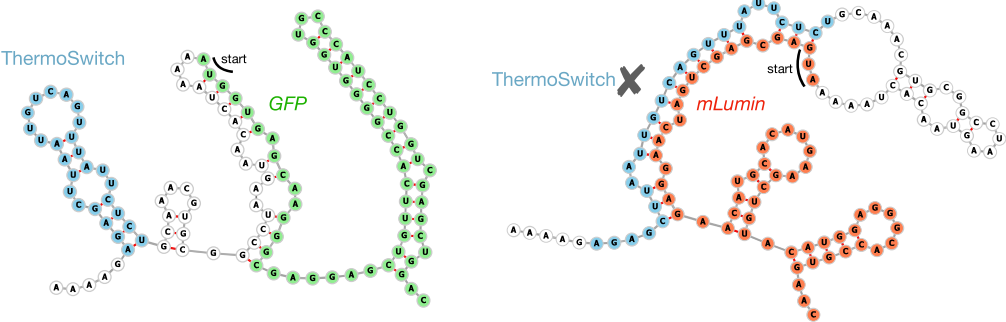

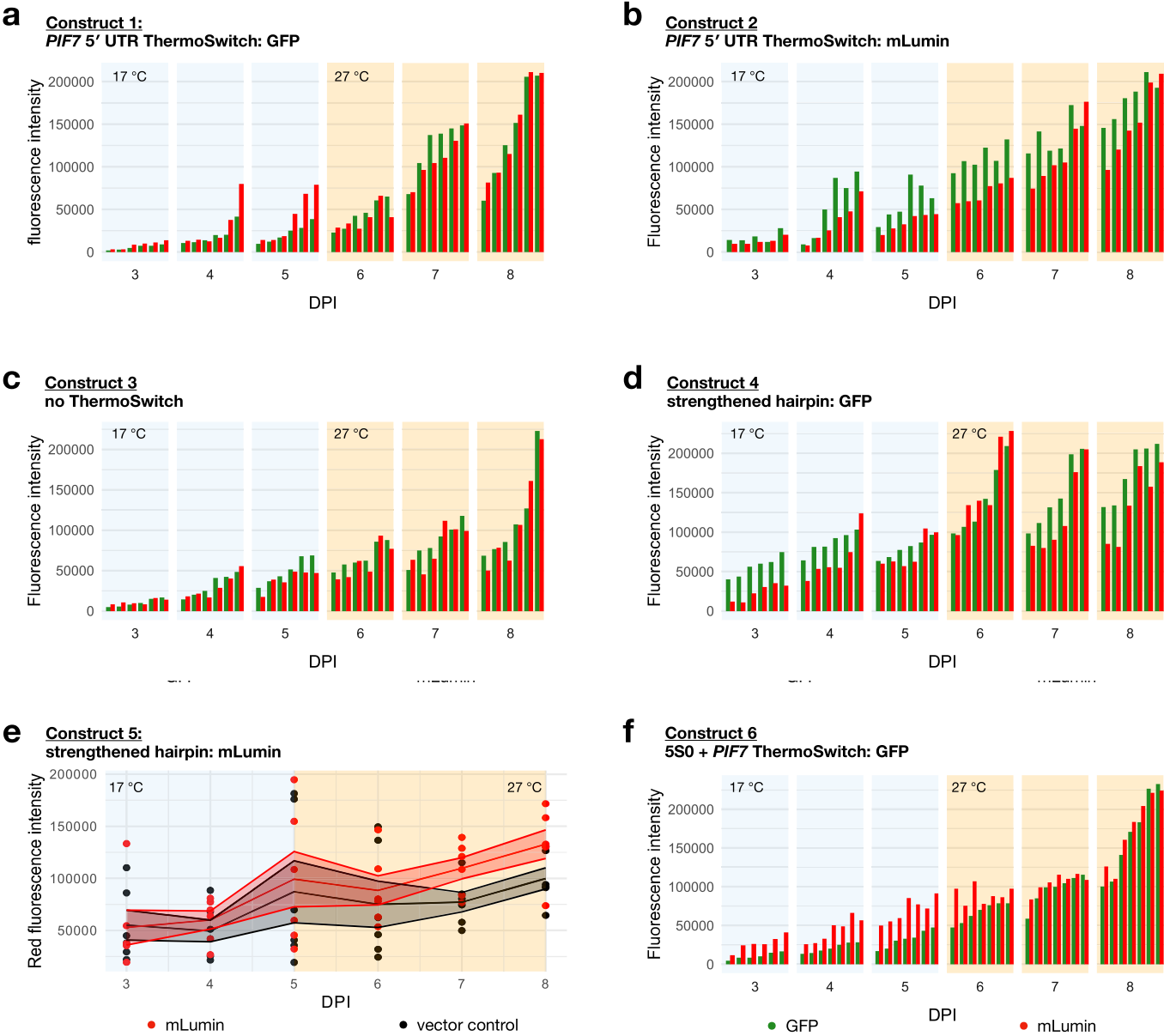

Supplement: Supplementary file 1 — Supplementary Material 1: Supplementary Figure 1 a and b, Leaves of N. benthamiana five days post infiltration and growth in a glasshouse (without temperature shift) photographed under UV light from the abaxial side and with white light from the adaxial side. VC = vector control group (pHRE). PIF7-str = PIF7 5′ UTR with a strengthened hairpin. Fluorescence measurement shown in Fig. 1e. c, Secondary structure of 60 nt of the 5′ UTR and 60 nt of the reporter CDS predicted by ViennaRNA RNAfold [19] at 17 °C and visualized using forna [18]. The ThermoSwitch hairpin sequence is coloured blue and the GFP or mLumin CDS is coloured green or red, respectively. Supplementary Figure 2. a–d, Blank-corrected fluorescence values of the leaf homogenate soluble fractions from the temperature shift experiments in Nicotiana benthamiana. Green bars show the 470-15/515-20 nm signal and red bars show the 585-15/630-20 nm signal. Black bars indicate the fluorescence of the area on the same leaf to which the empty vector was introduced. These values gave rise to the normalized fluorescence values in subfigures 2b–e. Mean of three technical replicates for each sample is shown. Blue and orange areas indicate growth at 17 °C or 27 °C, respectively, before the harvest. The temperature was shifted after the harvest on the fifth day. The fluorometry gain was adjusted individually for each experiment. f, Blank-corrected red (585-15/630-20 nm) fluorescence values of the leaf homogenate soluble fractions. Comparison of the vector control group (black) with the construct bearing strengthened hairpin upstream of mLumin (red). The ribbon shows the mean and the SEM. g, Blank-corrected fluorescence values of the leaf homogenate soluble fractions from the experiment using a combined 5S0 + ThermoSwitch 5′ UTR upstream of GFP. Black bars indicate the fluorescence of the area on the same leaf to which the empty vector was introduced. These values gave rise to the normalized fluorescence values in subfigure [file 13007_2026_1517_MOESM1_ESM.pdf]
